# Current Status of Circulating Tumor DNA Liquid Biopsy in Pancreatic Cancer

**DOI:** 10.3390/ijms21207651

**Published:** 2020-10-16

**Authors:** Miles W. Grunvald, Richard A. Jacobson, Timothy M. Kuzel, Sam G. Pappas, Ashiq Masood

**Affiliations:** 1 Department of Surgery, Rush University Medical Center, Chicago, IL 60612, USA; miles_w_grunvald@rush.edu (M.W.G.); richard_a_jacobson@rush.edu (R.A.J.); Sam_G_Pappas@rush.edu (S.G.P.); 2 Division of Hematology/Oncology and Cell Therapy, Rush University Medical Center, Chicago, IL 60612, USA; Timothy_Kuzel@rush.edu; 3 Rush Precision Oncology Program, Rush University Medical Center, Chicago, IL 60612, USA

**Keywords:** liquid biopsy, pancreatic cancer, cell free DNA, circulating tumor DNA

## Abstract

Pancreatic cancer is a challenging disease with a low 5-year survival rate. There are areas for improvement in the tools used for screening, diagnosis, prognosis, treatment selection, and assessing treatment response. Liquid biopsy, particularly cell free DNA liquid biopsy, has shown promise as an adjunct to our standard care for pancreatic cancer patients, but has not yet been universally adopted into regular use by clinicians. In this publication, we aim to review cfDNA liquid biopsy in pancreatic cancer with an emphasis on current techniques, clinical utility, and areas of active investigation. We feel that researchers and clinicians alike should be familiar with this exciting modality as it gains increasing importance in the care of cancer patients.

## 1. Pancreatic Cancer: The Need for a Novel Diagnostic 

Pancreatic ductal adenocarcinoma (PDAC) has an incidence of 13.1 cases per 100,000 persons in the United States, where it is currently the third leading cause of cancer mortality, and is expected to rise [1]. Five-year overall survival (OS) across all stages is a dismal 10% [1,2,3,4]. Early stage disease can be treated with curative intent, however, 5-year OS even in this case is 20–40% [5,6,7]. Poor survival in PDAC is attributed to advanced stage at presentation. Thus, in addition to the development of more effective treatment strategies for metastatic disease, the key to improve survival is early detection and implementation of curative-intent therapy.

Early detection of PDAC is limited by issues related to the tumor itself and the technology available for diagnosis. PDAC is often clinically silent in the early phases; only 10–20% of patients with PDAC are candidates for curative-intent therapy at time of diagnosis [8,9]. Presently, the United States Preventive Service Task Force (USPSTF) recommends against screening of the general population for PDAC owing to lack of an appropriate diagnostic assay. Available tests are neither sensitive nor specific for definitive diagnosis, and it is perceived that the harm of population level screening would outweigh the benefits of early detection [10,11].

The diagnosis of PDAC is currently confirmed via endoscopic ultrasound (EUS) with fine-needle aspiration for cytologic analysis [12,13]. Management is multimodal and depends chiefly on staging with EUS and radiographic cross-sectional imaging, often with computed tomography (CT) [8]. The decision to pursue or forgo surgical resection and curative intent therapy is a critical branch point in all treatment algorithms. Multidisciplinary tumor boards stratify patients into surgically resectable, borderline resectable, or unresectable subgroups based on the presence of distal disease and/or local tumor advancement [14]. Following treatment with either neoadjuvant chemotherapy, upfront surgery, or palliative intent systemic therapy, patients undergo serial evaluation with imaging and CA 19-9 analysis for surveillance in a curative setting or to ascertain response to therapies in a palliative setting [8,15,16,17].

**Figure 1:**
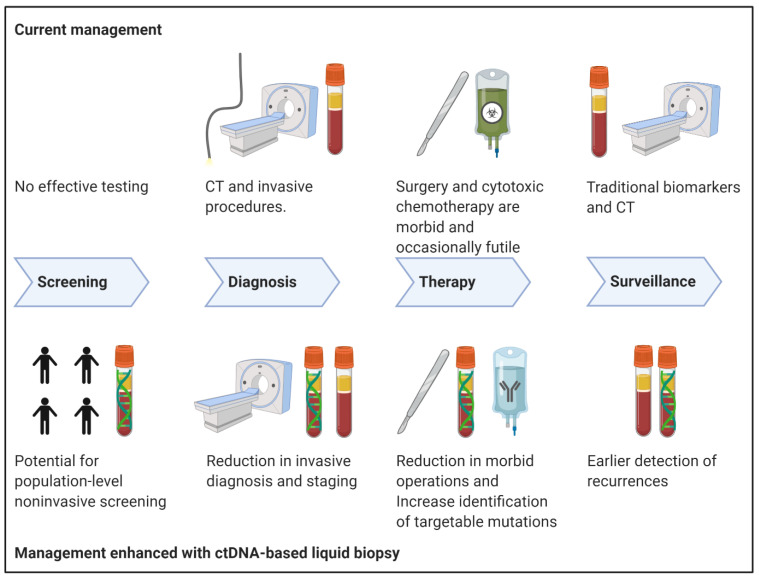
Potential impact of ctDNA-based liquid biopsy on multimodal management of pancreatic ductal adenocarcinoma (PDAC). Created with BioRender (BioRender.com, accessed 10 September 2020). CT, computed tomography.

Liquid biopsy is an emerging technology that permits the noninvasive sampling of tumor material in circulation [18]. It is under investigation for a number of gastrointestinal malignancies and is believed to offer immediate and theoretical advantages over current standards of care in several aspects of oncologic assessment (Figure 1) [19,20]. Our aim is to outline the present status of liquid biopsy as it relates to the management of PDAC with a focus on the use of cell free DNA (cfDNA) and circulating tumor DNA (ctDNA).

## 2. cfDNA and ctDNA in PDAC

The term “cfDNA” refers to free DNA, usually within the circulatory system, derived from both benign and malignant cells. “ctDNA” is more specific and refers to the tumor-derived portion of the cfDNA [18]. The principle behind peripherally detected cfDNA is that, as malignant and benign cells undergo apoptosis or necrosis, genetic material is released into circulation as either free nucleic acids or material that has remained encapsulated within the bounds of a partial or complete cell membrane [21,22,23,24,25]. The history of cfDNA is fairly recent; it was first isolated from human plasma in 1948 [26]. By 1965, a link between cfDNA and malignant processes had been established [27].

Liquid biopsy, broadly, uses one or more circulating biomarkers to assess a disease process. In the case of PDAC, much attention has been given to cfDNA, ctDNA, tumor-derived exosomes, and circulating tumor cells (CTCs) [28,29]. The ctDNA can provide clinicians information regarding PDAC as it harbors mutations or epigenetic characteristics specific to the patient’s cancerous process. The advancement of cfDNA and liquid biopsy-based cancer research has been largely dependent on parallel advances in oncogenetics and genomics, as these fields have characterized the key mutations that could be detected in peripheral blood samples.

Mutations present in the primary tumor, metastatic lesions, or both are detectable and sometimes distinguishable from one another. Mutations not detected on tissue biopsy because of intratumoral heterogeneity and sampling error may be detectable on a liquid biopsy. Low concordance between primary tumor and ctDNA collected from the periphery has been demonstrated [24]. Assessment of liquid biopsy derived genetic material has the potential to contribute to the diagnosis, staging, and surveillance of PDAC or potentially guide precision targeted therapy based on the specific mutation identified.

The identification and characterization of *KRAS* mutations as they relate to PDAC began in the 1980s. *KRAS* is often a founding mutation in pancreatic cancer, present in over 90% of primary tumors, and thus has become a primary target for analysis in ctDNA-based assays [30]. Additional, common mutations identified in the ctDNA of patients with PDAC include *CDKN2A*, *SMAD4*, and *TP53*. Further, actionable alterations in the *RTK-RAS-RAF* pathway including *BRAF* mutations, *ERBB2* mutations, and *FGFR1* amplifications are found in a smaller percentage of PDAC [31]. Tumors without KRAS mutations may harbor other mutations that can be detected with modern amplification and sequencing techniques [24].

### A Brief Mention of Exosomes

Exosomes are extracellular vesicles, typically between 50 and 150 nanometers in size, derived from cells (including cancer cells) that contain proteins and genetic material that may be isolated and analyzed. Theoretical advantages of exosome analysis in liquid biopsy are that exosomes have a longer circulating half-life than ctDNA and cancer cells are constantly producing exosomes so peripheral circulation capture may not be dependent on events such as cell necrosis, apoptosis, or invasion. These characteristics may allow for earlier detection [32,33,34].

Exosomes can be detected in many different fluids including effusions, ascites, blood, and saliva. Within the discipline of PDAC care, exosomes are being actively explored, but are not currently in widespread clinical use. Protein and genetic analysis may prove useful in diagnostics, screening, and targeted therapy [35,36,37].

## 3. Liquid Biopsy Techniques in PDAC

**Figure 2:**
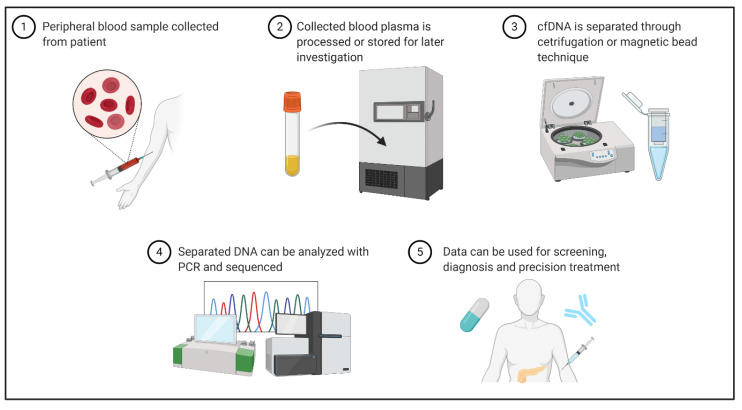
Sample collection, storage, DNA isolation, amplification and sequencing, and clinical application are important steps of ctDNA liquid biopsy. Created with BioRender (BioRender.com, accessed 10 September 2020). PCR, polymerase chain reaction.

cfDNA-based liquid biopsy is a multistep process involving sample collection, processing, and polymerase chain reaction (PCR)-based nucleic acid analysis. The overarching processes are well described. While laboratory protocols are generally standardized, some variation exists in isolation/purification, PCR, and sequencing (Figure 2) [29].

One important area of this process is the isolation and purification of the cfDNA. Two techniques have predominated this crucial part of cfDNA analysis, spin column or magnetic bead. At this juncture, the spin column technique is considered the gold standard, with slightly higher yields. DNA isolation may be one of the limiting factors in PDAC liquid biopsy as early disease frequently has exceptionally low levels of peripheral ctDNA, making a meaningful yield challenging [38].

Amplification/quantification follows the isolation/purification steps. In the case of PDAC, *KRAS* mutations are a common target, as over 90% of PDAC contains at least one *KRAS* mutation in tissue samples [39]. Of *KRAS*-mutated cancers, 90% will have one of four common polymorphisms: G12D, G12V, or G12R (40, 36, and 12 percent, respectively, for tissue samples) [40,41]. Various PCR protocols have been utilized including quantitative and droplet/digital PCR for amplification [42].

Sequencing can occur following amplification. Recently, high-throughput next generation sequencing (NGS)/massively parallel sequencing (MPS) has been utilized in ctDNA-based liquid biopsy [40,43,44,45]. Both entire ctDNA sequencing and targeted gene sequencing have been attempted [45,46]. This type of modality may be particularly useful in screening for actionable gene mutations and the advancement of precision oncology [47]. Success has been mostly demonstrated in patients with high tumor burden, and thus a higher percentage of ctDNA in circulation. Studies utilizing NGS have covered over 20,000 genes (and sometimes target specific genes); coverage (depth) has been variable, but 234× to 2227× has been reported in the literature [46,48].

CTCs and exosomes can be analyzed in a similar fashion to cfDNA. Flow cytometry, electron microscopy, or Western blot analysis can all be utilized in the identification of exosomes. However, unlike cfDNA, exosomes require ultracentrifugation for separation from interfering blood components [49]. Next, membranes are lysed and DNA/RNA can be isolated. In addition to analysis of nucleic acids, exosomes and CTCs can undergo quantification, protein analysis, and transcriptomics, providing additional information to researchers and clinicians [50].

## 4. Current Clinical Utility of cfDNA

### 4.1. ctDNA as a Screening Test

Screening for PDAC has the potential to improve outcomes if early stage disease can be detected; localized cancer has a five-year OS of 20% compared with metastatic PDAC’s 3% [2]. No PDAC screening tests are currently available for the average risk person and active investigation into the utility of liquid biopsy, specifically with ctDNA, is underway.

A meta-analysis including seven studies of ctDNA using various isolation protocols, gene loci, and epigenetic markers demonstrated a pooled sensitivity of only 0.64 [51]. The highest recorded sensitivity in an individual study was greater than 0.95 and the lowest was 0.27 [52,53,54,55,56]. The most widely accepted hypothesis underlying the low observed sensitivity is that, in early PDAC, there is an inadequate quantity of tumor necrosis necessary to release a detectable amount of peripheral ctDNA [30]. In the early stages of PDAC, there may only be one molecule of ctDNA per every 5 mL of plasma [51].

Another issue is the 20% rate of false positives derived from circulating *KRAS* mutations in patients with chronic pancreatitis [33]. On the other hand, used in combination with CA19-9 in a study of 47 patients, ctDNA was able to differentiate PDAC from chronic pancreatitis with a sensitivity of 98% and a specificity of 77% [57]. Multitarget screening tests may be more sensitive and have shown promise [58,59,60]. Advancement in techniques for increasing ctDNA yields will be necessary for the development of a ctDNA-based PDAC screening test. New research in epigenetics, specifically DNA methylation, has shown promise in improving sensitivity, however, larger and more comprehensive prospective studies are warranted [54,56,61,62].

### 4.2. ctDNA as a Diagnostic and Prognostic Test

Currently, diagnostic tests for PDAC include the combination of cross-sectional imaging and image-guided biopsy, usually an EUS guided fine-needle aspiration. These biopsies are invasive; unpleasant for the patient; and carry the risks of anesthesia, perforation, and infection. One of the endeavors of liquid biopsy would be to minimize the need for such invasive tests.

Compared with EUS, ctDNA offers increased convenience and lower risk. A recent meta-analysis demonstrated a pooled specificity for ctDNA in detection of PDAC of 0.92. This utility is limited in early disease, as mentioned previously. The current standard biomarker, CA19-9, has a specificity for PDAC of over 90% at certain cutoff values, thus, with current technology, ctDNA offers only a marginal if any improvement (regarding assessing whether or not PDAC is present) [52,63]. The use of ctDNA liquid biopsy in conjunction with traditional biomarkers has shown some promise in improving sensitivity and specificity in early PDAC [58]. However, if current trends continue and the diagnostic accuracy of ctDNA-based liquid biopsy continues to improve, one could envision a time when the need for invasive tissue biopsy of the primary lesion is obviated.

Potential advantages of ctDNA-based liquid biopsy are seen in its ability to prognosticate, as well as to diagnose actionable mutations in a minimally invasive fashion. Regarding its prognostic capabilities, current research has demonstrated that greater than 0.6% ctDNA was predictive of an OS of 6.3 months, while less than 0.6% had an OS of 11.7 months [24]. Peripherally detected *KRAS* mutations are a poor prognostic indicator at all stages of disease [64]. Mutated *KRAS* in the peripheral circulation has demonstrated worse OS (3 vs. 11 months in some studies) [65,66,67,68,69].

Regarding the diagnosis of actionable mutations for targeted therapy, ctDNA-based liquid biopsy has shown great promise. This is particularly important in the setting of metastatic disease where targeted therapies are being implemented for patients who have progressed while receiving standard of care regimens. It is thought that ctDNA has a higher concordance with metastatic lesions than primary tumor tissue [24,70,71]. Metastatic lesions may contain targetable mutations that are not present within the biopsy of the primary site. Liquid biopsy may diagnose these mutations, broadening treatment options and sparing additional biopsies for the sickest of PDAC patients. Within this context, ctDNA is being actively explored as a means to guide systemic treatment [72,73]. Common targetable mutations in metastatic PDAC include specific *KRAS* mutations, *BRAF*, and *ERBB2,* among others [73,74]. At present, this theoretical advantage has not yet translated into a quantifiable clinical benefit.

### 4.3. ctDNA in Assessing Resectability of Primary Tumor

The determination of resectability is key to the multidisciplinary management of PDAC. The principal determinants of resectability are (1) the degree of local mesenteric vascular involvement and (2) the presence of distant metastases [8]. EUS and pancreas protocol CT have an excellent diagnostic yield in evaluating the anatomic relationships to mesenteric vessels that dictate resectability, thus the role of ctDNA in this respect may be limited [75]. Higher levels of peripheral *KRAS* mutation have been associated with the presence of direct mesenteric vascular involvement, but with a low degree of sensitivity and specificity [65,66]. Portal venous sampling is feasible and has a higher ctDNA yield than peripheral blood, however, the relationship of portal ctDNA levels to local vascular invasion is unknown. Further, portal venous sampling is invasive and negates the theoretical advantage of a noninvasive peripheral blood liquid biopsy.

On the other hand, room for improvement exists in the detection of systemic disease that would preclude attempts at resection. The 80% incidence of distant metastases within 5 years of an R0 resection implies that no contemporary test adequately detects systemic disease in the preoperative period [76,77]. The majority of metastases occur in the liver or peritoneal surface, where sub-centimeter malignant implants are difficult to detect radiographically [22]. Current staging protocols involve chest and pelvic CT, magnetic resonance imaging (MRI) or positron emission tomography (PET) of the abdomen to evaluate indeterminant liver lesions, and in some cases, staging laparoscopy. Elevated traditional biomarkers such as CA19-9 are predictive of occult metastases, albeit with a false positive rate up to 47% [78]. A study of 1001 patients since 2000 demonstrated that even laparoscopy, which requires operating room time and general anesthesia, missed between 10 and 30% of occult metastases identified during open surgery, without mention of early recurrences in resected patients [79]. Discordance between mutations detected in ctDNA versus tissue biopsy may indicate the presence of radiographically occult metastases. The fact that ctDNA is more concordant with mutations identified in tissue biopsy from metastatic lesions than the primary tumor supports this [24]. However, intratumoral heterogeneity and sampling error in the primary tissue biopsy partially account for this observation [80].

ctDNA-based liquid biopsy has the potential to assist in the detection of occult metastatic disease. This could spare patients the morbidity of an operation from which they would reap no benefit. A study of 112 patients demonstrated that those with resectable disease had both a lower percent ctDNA and fewer genomic alterations in ctDNA than patients with unresectable disease [24,81].

As *KRAS* is mutated in over 90% of PDAC and is reliably detectable in inexpensive assays, ctDNA-based liquid biopsy designed to detect mutations at this locus for staging purposes can be a feasible strategy if confirmed in a prospective large study [82]. A study of 151 patients demonstrated peripherally detected *KRAS* mutations in 58.9% of patients with radiographic distant organ metastasis compared with 18.2% of patients without them. In the same study, only 9 out of 108 (8.3%) patients with successfully resected disease had peripherally detected *KRAS* mutations. Only 4.6 percent of patients with a negative preoperative ctDNA-based liquid biopsy insuffered distant recurrences within 6 months of surgery, compared with 35% in a separate study with radiographically defined resectable disease, but no liquid biopsy testing [83,84]. Thus, radiographic staging and then further stratification with ctDNA may optimize the selection of patients who would benefit from surgical resection. A study of 23 patients with detectable ctDNA prior to surgical resection demonstrated that 12 converted to ctDNA-negative in the postoperative period. Of these 12, median recurrence free survival was inferior to those with undetectable preoperative ctDNA at 12.2 months compared with over 38 months in the preoperatively negative ctDNA group [85]. Early data have demonstrated that patients who have undergone neoadjuvant chemotherapy and are ctDNA negative have an 80% chance of an R0, node negative resection compared with those with who are ctDNA positive, having only a 38% chance of an R0, node negative operation [86]. Taken together, these data indicate that patients with radiographically resectable disease may fall, broadly, within two subsets: those who are ctDNA positive with worse outcomes and those who are ctDNA negative with better outcomes [87,88,89].

Preoperative ctDNA positivity clearly indicates a high risk of postoperative recurrence; however, further prospective research is required to determine whether it is a hard indicator of surgical futility. Ultimately, based on the combined results of preoperative ctDNA and traditional imaging modalities, patients could either be spared the morbidity of a laparotomy in cases where it is futile, or referred for neoadjuvant therapy and restaged.

### 4.4. Surveillance

Recurrence after curative-intent therapy is high in PDAC. One study of patients with early stage, resectable disease demonstrated 35% recurrence at 1-year follow up, while 1-year recurrence rates over 80% have been observed in patients with borderline resectable disease [84,90]. Early detection of recurrences is key to initiating appropriate systemic therapy. Surveillance for recurrence or progression of PDAC involves traditional tumor markers such as CA19-9 and cross-sectional imaging. ctDNA may be superior to current techniques in the sense that it offers earlier detection and the opportunity to guide targeted therapy [74]. The short half-life of ctDNA in circulation (measured in hours) is ideal for assessing response to therapy in shorter intervals [91].

In 10 patients who recurred after curative-intent therapy, ctDNA-based liquid biopsy was able to detect disease progression at 3.1 months, compared with 9.6 months with standard CT-based surveillance [48]. Postoperatively detectable ctDNA had a 100% positive predictive value for disease recurrence with a sensitivity of 57%. While 13 of 13 patients with postoperatively detectable ctDNA relapsed, only 10 of 22 patients with undetectable postoperative ctDNA had recurrences at 38 months post-resection [92]. CA19-9 has traditionally been used as a surveillance tool, but has its limitations, namely low sensitivity and the fact that 5–10% of the population are incapable of producing CA19-9, rendering it useless in these patients [93,94]. ctDNA can be used for monitoring throughout treatments including surgery and chemotherapy [95,96,97,98,99].

Future application of ctDNA in surveillance is an exciting area of investigation. Further incorporating ctDNA guided surveillance in large prospective studies may aid in the development of risk-adjusted treatment strategies for adjuvant chemotherapy. For instance, a less aggressive chemotherapy regimen may be appropriate for patients who are ctDNA negative, sparing them from unpleasant toxicities, whereas patients with persistent ctDNA on completion on adjuvant therapy may need close surveillance so that recurrence is detected early to improve the clinical outcomes.

### 4.5. Clinical Utility Of Exosomes

Exosomes remain an area of active exploration. Both genetic and protein components of exosomes have been assessed for utility in PDAC. In particular, glypican 1 expression has been proposed as an adjunct for PDAC diagnosis [35,36,100]. PDAC-associated *KRAS* mutations have been reliably identified in exosome derived RNA/DNA [33,36,101]. However, similar to cfDNA, early detection remains challenging and limits utility as a screening tool [33]. There remains a paucity of research on the utility of exosomal analysis for tracking response to treatment or as a prognostic tool in PDAC.

## 5. Ongoing Clinical Trials and the Future of Cfdna in Pdac

While not the standard of care at present, cfDNA-based liquid biopsy in its current form can contribute to management of PDAC and a wide range of new indications for its use are under development. Current trials are exploring screening, prognostics, precision oncology, and targeted therapy. Table 1 lists current trials registered with clinicaltrials.gov. cfDNA-based liquid biopsy is an exciting field that has applications for multiple cancers, the scope of which is not covered in this review, yet advances in other fields will likely come with parallel progress in liquid biopsy for PDAC.

## 6. Conclusions

PDAC remains a deadly and challenging cancer for patients and clinicians, respectively. There are opportunities for improvement in screening, treatment guidance, and surveillance. The use of ctDNA as a liquid biopsy is an area of active and fruitful investigation.

One of the most valuable areas in which ctDNA can be utilized is as a guide toward or away from surgical resection based on detection of ctDNA, typically by the presence of mutated KRAS. Microscopic metastases have been a persistent problem in the care of PDAC, and ctDNA may be the tool that helps identify patients with this condition prior to receiving unwarranted therapy. Thus, for radiographically resectable disease, the utility of ctDNA lies in the identification of patients appropriate for neoadjuvant therapy, and in sparing certain patients the morbidity of major abdominal surgery in cases where it would be futile. Multiple small studies have demonstrated the utility of ctDNA as a tool for monitoring response to different treatments. As novel, targeted therapies continue to emerge, the role of genetic assessment for these targets will play an ever-larger role in cancer care. ctDNA analysis with NGS seems to be a promising field and should be further explored in the setting of PDAC.

ctDNA-based liquid biopsy has potential to be implemented in multiple phases of care in patients with pancreatic cancer if confirmed in large prospective studies. We anticipate adoption into the clinician’s tool kit over the next several years once current clinical trials come to conclusion.

## Figures and Tables

**Table 1:** Current trials registered with clinicaltrials.gov exploring liquid biopsy with cfDNA in pancreatic cancer.

Trial Number	Trial Name	ctDNA Liquid Biopsy Focus/Goals	Study Type	Estimated Completion	Recruitment Status
NCT02079363	DNA Promoter Hypermethylation as a Blood Based Maker for Pancreatic Cancer	Assessment of hypermethylation as diagnostic, prognostic, and recurrence marker	Prospective Observational Cohort	January 2018	Unknown
NCT03524677	Mutation of K-RAS, CDKN2A, SMAD4 and TP53 in Pancreatic Cancer: Role of Liquid Biopsy in Preoperative Diagnosis	Assessment of four ctDNA mutations’ impact on preoperative staging and progression	Prospective Observational Cohort	January 2020	Recruiting
NCT02934984	Circulating Cell-free Tumor DNA (ctDNA) in Pancreatic Cancer	ctDNA as a tool for surveillance/screening for recurrence	Prospective Observational Cohort	January 2021	Recruiting
NCT04246203	Prognostic Role of Circulating Tumor DNA in Resectable Pancreatic Cancer (PROJECTION)	ctDNA as prognostic indicator in patients with radiographically resectable cancer	Prospective Observational Cohort	March 2025	Not yet recruiting
NCT04241367	Verification of Predictive Biomarkers for Pancreatic Cancer Treatment Using Multicenter Liquid Biopsy	Assessment of ctDNA KRAS mutation on outcomes in pancreas cancer. Will also assess ctDNA for specific gene targets	Prospective Observational Cohort	December 2025	Recruiting
NCT04176952	PRIMUS002: Looking at 2 Neo-adjuvant Treatment Regimens for Resectable and Borderline Resectable Pancreatic Cancer	ctDNA liquid biopsy as a means to stratify response to a chemotherapy regimen (secondary outcome)	Phase 2 Clinical Trial	December 2023	Recruiting
NCT04484636	PLATON - Platform for Analyzing Targetable Tumor Mutations (Pilot-study) (PLATON)	Assessing peripheral blood samples for targetable mutations in multiple gastrointestinal malignancies	Prospective Observational Cohort	June 2021	Not yet recruiting
NCT03334708	A Study of Blood Based Biomarkers for Pancreas Adenocarcinoma	Determination of sensitivity and specificity of ctDNA for the diagnosis of early stage pancreatic cancer	Prospective Observational Cohort	October 2021	Recruiting
NCT03568630	Blood Markers of Early Pancreas Cancer	Identification of blood markers of early pancreas cancer	Prospective Observational Cohort	July 2023	Recruiting

## Abbreviations

PDAC	Pancreatic Ductal Adenocarcinoma
USPSTF	United States Preventative Service Task Force
OS	Overall Survival
EUS	Endoscopic Ultrasound
cfDNA	Cell Free Deoxyribonucleic Acid
ctDNA	Cell Free Tumor Deoxyribonucleic Acid
CTCs	Circulating Tumor Cells
PCR	Polymerase Chain Reaction
NGS	Next Generation Sequencing
